# Correlation Between Maximal Eccentric and Isometric Multi-Joint Lower-Extremity Strength and Vertical Jumping Performance in Young Adults

**DOI:** 10.3390/muscles3040034

**Published:** 2024-12-03

**Authors:** Joonsun Park, Cassidy Weeks, Brennan J. Thompson, Talin Louder

**Affiliations:** 1 Kinesiology and Health Science Department, Utah State University, Logan, UT 84322, USA; joonsun.park@usu.edu (J.P.); brennan.thompson@usu.edu (B.J.T.); 2 Movement Research Clinic, Sorenson Legacy Foundation Center for Clinical Excellence, Utah State University, Logan, UT 84322, USA; 3 School of Medicine, University of Colorado Denver–Anschutz Medical Campus, 13121 East 17th Ave., Aurora, CO 80045, USA; weeks.cassi@gmail.com

**Keywords:** countermovement jump, depth jump, ground reaction force, isokinetic, dynamometry, stretch shortening cycle

## Abstract

Maximal eccentric (MES) and isometric (MIS) muscle strength may enhance vertical jump performance by facilitating preloading and reducing energy loss during the eccentric (ECC) phase of the stretch-shortening cycle (SSC). However, the contributions of ECC and isometric (ISO) strength to the countermovement (CMJ) and depth jump (DJ) remain unclear due to variability in assessment methods (e.g., dynamometry, isometric mid-thigh pull) and the limited range of metrics examined in prior research. The aim of this study was to assess correlations between multi-joint lower extremity MES and MIS, obtained using a seated multi-joint isokinetic dynamometer, and 13 vertical ground reaction force (GRF) measures derived from the performance of three maximal effort DJs and CMJs. Twenty-five healthy young adults participated in this study (age = 21.9 ± 2.9 years). Pearson *r* correlation coefficients were used to assess the statistical significance (α = 0.05) of the relationships between absolute (N) and body mass normalized (BN) maximal strength measures and vertical jumping metrics. Moderate-to-strong positive correlations were identified between MES and MIS with broad performance metrics in CMJ and DJ, including reactive strength index (*r* = 0.45–0.53, *p* < 0.05), modified reactive strength index (*r* = 0.41–0.62, *p* < 0.05), and jump height (*r* = 0.59–0.75, *p* < 0.05). Moderate-to-strong positive correlations were also observed between MES and MIS with CON work (*r* = 0.58–0.71, *p* < 0.05) and CON peak power (*r* = 0.44–0.71, *p* < 0.05) for both the CMJ and DJ. In contrast, moderate-to-strong negative correlations were observed between MES and MIS with ECC work (*r* = 0.42–0.62, *p* < 0.005) and ECC peak power (*r* = 0.45–0.60, *p* < 0.05). These findings suggest that enhanced neuromuscular efficiency and joint stiffness in stronger musculature reduce energy absorption during the eccentric phase, minimizing mechanical deformation and preserving elastic energy for concentric propulsion. Combined, MES and MIS optimize force application, energy utilization, and control, which are crucial for maximizing jump height. These findings underscore the role of MES and MIS in influencing jumping performance across both the ECC and CON phases of the SSC. This insight is valuable for practitioners designing training programs aimed at improving vertical jumping ability.

## 1. Introduction

The rapid sequence of eccentric (ECC), isometric (ISO), and concentric (CON) muscle actions is collectively known as the stretch-shortening cycle (SSC) [1]. SSC actions involve elongating a pre-activated skeletal muscle during the ECC phase, followed by promptly engaging the muscle in a forceful CON action [1]. This cycle is viewed as a fundamental skeletal muscle action that enhances CON muscle performance through a combination of passive and neuroactive mechanisms when explosive athletic movements are executed fluidly and sequentially during functional movement tasks [1]. More specifically, the SSC enhances the CON force and power generation capacity of skeletal muscle, surpassing what can be achieved through an isolated CON action [1].

During the ECC phase of the SSC, there is a temporal window for force potentiation, leading to increased preload during the subsequent CON phase. This increased preload results from a combination of potential energy utilization within the series and parallel elastic components of skeletal muscle and the potentiation of alpha motor neurons via spinal reflex circuitry [2]. Preload achieved during the ECC phase is influenced by the duration of the amortization phase, which is the time required to transition from the ECC to CON phase. For instance, minimizing the duration of both the ECC and amortization phases is suggested to enhance preload and thereby contribute to superior performance in developing maximal CON power [3].

Vertical jumping movements, such as the countermovement jump (CMJ) and depth jump (DJ), rely heavily on the SSC and are among the most common movements chosen for investigating and assessing the SSC ability of lower-extremity skeletal muscle [4,5]. The ECC phase of vertical jumping involves a rapid lowering of the whole-body center of mass and is suggested to directly impact jumping mechanics [3]. This phase prepares the muscles for a more powerful CON action by optimizing force generation and energy storage [1]. The ECC phase of jumping is associated with the potentiation of alpha motor neurons via muscle spindle afferents and increased stiffness of the fascicles, leading to less fascicle lengthening and more tendinous lengthening [1]. Consequently, this results in heightened alpha motor neuron activity beyond what is achieved through descending voluntary central nervous system activity, and it optimizes the muscle fiber length, thereby generating greater force during the CON phase [6,7,8].

Previous research has demonstrated that jumping techniques involving a rapid ECC phase result in greater vertical jump heights (JHs) compared to those that isolate CON skeletal muscle action, such as squat jumps [9,10,11]. This underscores the fundamental role of the ECC phase of the SSC in facilitating jumping performance. It also highlights the necessity of understanding the extent to which maximal muscular strength influences both the ECC and subsequent CON phases of jumps that utilize the SSC. Maximal eccentric strength (MES) and maximal isometric strength (MIS) are widely recognized metrics for assessing maximal muscular strength. MIS measures the maximum tension a skeletal muscle can sustain in a static hold, where any further load increase would result in an eccentric action. MES, on the other hand, measures the maximum tension a muscle can tolerate while lengthening, typically at a controlled joint angular velocity.

A recent meta-analysis indicates that only a limited number of studies have explored the correlation between MES and vertical jumping performance [12]. For instance, Bridgeman et al. [13] utilized an Exerbotics squat machine (eSQ; Exerbotics, LLC, Tulsa, OK, USA) to measure MES during two repetitions of a squat, with the ECC phase lasting for 4 s each. They observed a significant positive correlation between MES and CMJ JH in strength trained athletes (*r* = 0.73, *p* <0.001). Additional findings from the literature include significant positive correlations between isokinetic eccentric force of the quadriceps (60°/s, 180°/s) and CMJ JH in a sample of acrobatic skydivers (*r* = 0.658–0.737, *p* = 0.004–0.011) [14]. Furthermore, a significant positive correlation has been observed between isoinertial eccentric back squat force and reactive strength indices (RSI) corresponding with DJs performed from a height of 0.50 m by competitive team sport and track-and-field athletes (*r* = 0.60, *p* <0.050) [15].

Prior literature examining correlations between MIS and CMJ performance is more extensive but presents mixed and inconsistent findings [16,17,18,19]. Using the ISO mid-thigh pull (IMTP) test to measure MIS, Dos Santos et al. [16] observed a non-significant positive correlation between MIS and CMJ JH (ρ = 0.441, *p* = 0.441), and a significant positive correlation between MIS and CMJ RSI-modified (RSImod; ρ = 0.389, *p* = 0.050). Similarly, Merrigan et al. [17] found significant correlations between IMTP MIS, CMJ JH (*r* = 0.460), and CMJ RSI-modified (*r* = 0.350). However, other investigations reported non-significant correlations between IMTP MIS and CMJ JH (*r* = −0.020–0.276) [18,19]. The inconsistency in findings likely stems from multifaceted influences, including differences in neuromuscular control across varied populations or inconsistencies in the methodologies used for IMTP or CMJ assessments.

To our knowledge, no study has examined the correlations between MES and MIS with CMJ and DJ ground reaction force (GRF) metrics within a single study. Establishing these relationships within a single study is crucial for distinguishing the contributions of different types of lower-extremity strength (i.e., MES vs. MIS) to vertical jumping performances that require different jumping techniques. For example, Nicol et al. [20] suggest that relationships derived from metrics of vertical jumping performance are task-dependent, as various jumping techniques, such as the CMJ and DJ, involve the SSC to varying extents and differ in terms of mechanical demand and neuromotor complexity [20].

Additionally, a significant portion of prior research has assessed maximal strength using either the IMTP test or single-joint isokinetic dynamometry. However, there has been notable variability in the testing protocols employed in studies utilizing the IMTP, which limits the generalizability of prior findings. Furthermore, considering the complexity of multi-joint coordination involved in vertical jumping, adopting a multi-joint approach for lower-extremity strength assessment may enhance the specificity of correlations obtained from MES, MIS, and GRF metrics of CMJ and DJ performance. Utilizing a seated dynamometer, such as the Eccentron (BTE Technologies, Inc., Hanover, MD, USA) addresses this gap in the current literature by providing controlled methods for assessing both multi-joint isokinetic eccentric and isometric strength.

Lastly, previous studies have generally focused on correlating lower-extremity strength with a limited subset of CMJ and DJ metrics. Therefore, there is a current need to assess correlations using a comprehensive array of CMJ and DJ metrics. While methods for deriving various CMJ metrics, such as rate of GRF development (RFD), amortization, work, power, and energy, have been established [21], similar measures from the DJ have been historically challenging to obtain. However, recent advancements in valid single-force platform methods [20,22] now make it feasible to estimate these measures. Thus, to address current gaps in the literature, the purpose of this study was to evaluate and contrast correlations between lower-extremity multi-joint MES and MIS, obtained through a multi-joint isokinetic dynamometer, and 13 vertical GRF measures derived from CMJs and DJs in young adults.

## 2. Results

Descriptive statistics are presented in Table 1 and Table 2.

### 2.1. MES and CMJ

Significant moderate-to-strong positive correlations were observed between absolute MES and JH, time to take-off (TTT), RSImod, amortization time (AmorT), CON work (ConW), and CON peak power (ConPP; *r* = 0.41–0.63, *p* = <0.001–0.044; See Table 3). Significant moderate negative correlations were observed between absolute MES and ECC work (EccW) and ECC peak power (EccPP; *r* = −0.56–−0.46, *p* = 0.003–0.021; See Table 3). No significant correlations were observed between absolute MES and unloading RFD (UnRFD), ECC RFD (EccRFD), amortization force (AmorFz), mean CON force (MeanConFz), or peak CON force (PeakFz; *r* = −0.07–0.34, *p* = 0.095–0.746).

Significant moderate-to-strong correlations were observed between body mass normalized (BN) MES and JH, RSImod, AmorFz, ConW, ConPP, and PeakFz (*r* = 0.45–0.65, *p* = <0.001–0.024; See Table 3). Significant moderate negative correlations were observed between BN MES and EccW and EccPP (*r* = −0.45–−0.46, *p* = 0.021–0.023; See Table 3). No significant correlations were observed between BN MES and TTT, UnRFD, EccRFD, AmorT, or MeanConFz (*r* = 0.15–0.30, *p* = 0.093–0.469).

### 2.2. MIS and CMJ

Significant moderate-to-strong positive correlations were observed between absolute MIS and JH, TTT, RSImod, AmorT, AmorFz, ConW, and ConPP (*r* = 0.43–0.75, *p* = <0.001–0.030; See Table 3). Significant moderate negative correlations were observed between absolute MIS and EccW and EccPP (*r* = −0.57–−0.51, *p* = 0.003–0.009; See Table 3). No significant correlations were observed between absolute MIS and UnRFD, EccRFD, MeanConFz, or PeakFz (*r* = 0.04–0.35, *p* = 0.089–0.853).

Significant moderate-to-strong positive correlations were observed between BN MIS and JH, RSImod, AmorFz, ConW, ConPP, and PeakFz (*r* = 0.47–0.75, *p* = <0.001–0.017; See Table 3). Significant moderate negative correlations were observed between BN MIS and EccW and EccPP (*r* = −0.48–−0.45, *p* = 0.016–0.023; See Table 3). No significant correlations were observed between BN MIS and TTT, UnRFD, EccRFD, AmorT, or MeanConFz (*r* = 0.05–0.28, *p* = 0.193–0.816).

### 2.3. MES and DJ

Significant moderate-to-strong positive correlations were observed between absolute MES and JH, RSI, ConW, and ConPP (*r* = 0.50–0.65, *p* = <0.001–0.014; See Table 4). No significant correlations were observed between absolute MES and ground contact time (GCT), EccRFD, EccW, EccPP, AmorT, AmorFz, MeanConFz, PeakFz, or peak force reduction (PFR) (*r* = −0.4–0.4, *p* = 0.050–0.868; See Table 4).

Significant moderate-to-strong positive correlations were observed between BN MES and JH, ConW, and ConPP (*r* = 0.44–0.59, *p* = 0.002–0.003; See Table 4). Significant moderate negative correlations were observed between BN MES and EccW and EccPP (*r* = −0.62–−0.46, *p* = <0.001–0.023; See Table 4). No significant correlations were observed between BN MES and GCT, RSI, EccRFD, AmorT, AmorFz, MeanConFz, PeakFz, or PFR (*r* = −0.29–0.4, *p* = 0.052–0.987; See Table 4).

### 2.4. MIS and DJ

Significant moderate-to-strong positive correlations were observed between absolute MIS and JH, RSI, ConW, and ConPP (*r* = 0.53–0.67, *p* = <0.001–0.008; See Table 4). A significant moderate negative correlation was observed between absolute MES and EccPP (*r* = −0.45; *p* = 0.029; See Table 4). No significant correlations were observed between MIS and GCT, EccRFD, EccW, AmorT, AmorFz, MeanConFz, PeakFz, or PFR (*r* = −0.23–0.40, *p* = 0.050–0.674; See Table 4).

Significant moderate-to-strong positive correlations were observed between BN MIS and JH, RSI, ConW, and ConPP (*r* = 0.45–0.62, *p* = 0.001–0.038; See Table 4). Significant moderate-to-strong negative correlations were observed between BN MES and EccW and EccPP (*r* = −0.6–−0.42, *p* = 0.002–0.040; See Table 4). No significant correlations were observed between BN MIS and GCT, EccRFD, AmorT, AmorFz, MeanConFz, PeakFz, or PFR (*r* = −0.22–0.31, *p* = 0.147–0.993; See Table 4).

## 3. Discussion

The purpose of this study was to investigate the relationships between lower-extremity multi-joint MES and MIS with various vertical GRF variables measured during maximal effort CMJs and DJs in healthy young adults. Our findings showed significant moderate-to-strong positive correlations between MES and MIS metrics and various aspects of jumping performance. We also found significant moderate-to-negative correlations between MES and MIS metrics with EccW and EccPP.

Reactive strength indices serve as comprehensive assessments of CMJ and DJ performance. We found significant moderate-to-strong positive correlations between all MES and MIS metrics and RSImod and RSI scores, with the exception of BN MES and DJ RSI. These correlations likely stem from the strong positive relationships observed between MES and MIS metrics and JH in both CMJ and DJ. Additionally, our findings on the positive correlations between MES and CMJ JH and DJ RSI are in line with existing literature [12,13,14,15]. Furthermore, our findings regarding the significant positive correlations between MIS, RSImod, and CMJ JH are also partially supported by previous studies [16,17,18,19].

MES, which involves the controlled lengthening of muscles under load, allows an athlete to effectively decelerate their body during the downward phase of a jump. This controlled deceleration is essential for building elastic energy, which is stored in the muscles and tendons and released during the upward, concentric phase of the jump [23,24,25,26]. Thus, greater MES may allow for greater energy storage, which can increase the force and speed of the propulsion phase, ultimately leading to a higher jump. MIS may be useful in stabilizing the body during the transition between eccentric and concentric phases in jumping [27]. By providing a stable foundation during this critical point, MIS could feasibly enable a faster and more efficient change from descending to ascending motion, maximizing the transfer of stored energy. Together, MES and MIS provide a robust combination for peak force application, energy utilization, and control, all of which are key to maximizing JH.

JH is the final product of the CON phase of the SSC, where agonist muscles act concentrically to generate force that is applied by the feet to the ground as mechanical impulse. We found significant positive correlations between all MES and MIS metrics and both ConW and ConPP across CMJ and DJ. ConW and ConPP are directly related to jump height because they determine the mechanical energy imparted to the body during the propulsion phase of a jump [28]. Mentioned previously, MES allows for greater force absorption and elastic energy storage during the lengthening phase, while MIS stabilizes the transition phase, minimizing energy dissipation and enabling efficient energy transfer. Additionally, eccentric and isometric strength enhance the force–velocity relationship [29] and neuromuscular recruitment, particularly of fast-twitch fibers, enabling greater force production and coordination during rapid, dynamic movements [30]. In depth jumps, where high impact forces increase eccentric demands, MES and MIS are crucial for energy absorption and propulsion. Enhanced stiffness in the musculotendinous unit, resulting from eccentric and isometric conditioning, further reduces energy loss and improves energy transfer efficiency [23,24,25,26]. Collectively, these factors underline the importance of MES and MIS in maximizing concentric metrics of jump performance.

It would be reasonable to expect that MES and MIS metrics would show a stronger and more consistent correlation with EccW and EccPP compared to the same CON GRF parameters in both CMJ and DJ performances. This expectation was supported for CMJs but not for DJs. For CMJ, significant moderate-to-strong negative correlations were observed between all MES and MIS metrics and both EccW and EccPP. However, for DJ, significant negative correlations were only evident when MES and MIS were normalized to body mass. Interestingly, for both DJ and CMJ, the negative correlations suggest that greater levels of BN MES and MIS are associated with reduced EccW and EccPP. These findings may reflect enhanced neuromuscular efficiency [30] and joint stiffness [31]. For instance, stronger musculature resists excessive lengthening during the eccentric phase, reducing mechanical deformation and energy absorption. This shift in energy demand from active muscle contraction to tendon elasticity minimizes eccentric work while preserving elastic energy for concentric propulsion [23,24,25,26]. Additionally, increased joint stiffness and faster stretch-shortening cycle transitions shorten the eccentric phase duration, further decreasing eccentric power output. These adaptations reflect a more efficient energy transfer mechanism, allowing stronger individuals to redirect energy toward concentric work and power generation, thereby optimizing jump performance.

Interestingly, the results indicate that MES and MIS may influence the temporal characteristics of CMJ performance. Specifically, we observed significant moderate positive correlations between absolute MES and MIS metrics with total TTT and AmorT. In contrast, no significant correlations were found between BN MES and MIS metrics and these temporal measures. The significant relationships with absolute strength metrics suggest that greater MES and MIS may enhance CMJ JH by enabling a deeper countermovement stretch of the agonist muscles or by increasing the time available for force application during the amortization and concentric phases. This extended time likely facilitates greater mechanical impulse generation, leading to increased vertical velocity and, consequently, higher CMJ JH. In depth jumping DJ, however, no significant correlations were observed between MES and MIS metrics and GCT or AmorT. This absence of correlation may reflect the unique mechanical and neuromotor demands of DJ compared to CMJ, such as the emphasis on rapid force application and shorter contact times [1,20].

The DJ technique differs from CMJ in that it begins with dropping from a set height, requiring the absorption of landing impact momentum before performing a maximal vertical jump. Consequently, the DJ places greater ECC stretch loads on muscles and tendons compared to the CMJ and requires a more sophisticated contribution of neuromotor control pathways [1,4,20]. Moreover, prior research has demonstrated that broad metrics of CMJ and DJ performance, such as RSImod and RSI, are not directly interchangeable correlates [20]. The unique characteristics of each jumping technique help explain the inconsistencies in correlations observed in this study. PFR is a variable measured exclusively for the DJ and has been used previously as a metric for SSC fatigue and reactive capacity [32]. We observed non-significant weak negative correlations between all MES and MIS metrics and PFR. A negative relationship with PFR is considered advantageous for SSC performance and jumping ability, as it indicates reduced force relaxation (e.g., Golgi tendon inhibition) following high-impact landing forces. This reduction in force relaxation helps maintain higher force output through the CON phase of the DJ, potentially leading to greater JH.

While this study is among the first to assess the correlation between MES, MIS, and a wide array of GRF metrics across both CMJ and DJ performances—and provides valuable insights into the association between MES, MIS, and enhanced jumping performance—it is not without limitations. The sample size in this investigation was insufficient to detect statistical significance in weak correlations, such as those observed with PFR and other metrics. While the term “weak” might suggest that the findings are inconsequential, it is arguable that even weak associations can offer meaningful insights into the relationship between maximal strength and specific metrics of CMJ and DJ performance. Future investigations should consider increasing study power by enrolling a larger number of participants. The sample of participants in this study was relatively homogeneous, consisting primarily of recreationally active young adults. Therefore, future research should explore whether these findings are replicable across different populations, including individuals of varying ages, athletic backgrounds, and levels of physical activity participation. Also, MES and MIS were assessed with participants in a seated position, whereas jumps were performed in a standing position. This positional difference may lead to variations in muscle activation patterns and engagement levels of key muscle groups. The seated position may limit the activation of muscles that are more engaged in standing movements, potentially affecting the correlations observed in the present study. Lastly, this study employed cross-sectional research methods. While Papadopoulos et al. [33] reported that an 8-week ECC exercise program led to a significant increase in MES (13.6 ± 3.2%) alongside concurrent improvements in DJ JH (*p* < 0.001), further longitudinal research is necessary to better understand the contributions of MES and MIS to the performance of various jumping techniques.

## 4. Materials and Methods

### 4.1. Participants

In total, 25 young adults (17 male, 8 female) volunteered to participate in the investigation (mean ± standard deviation; age: 21.9 ± 2.9 years; height: 173.9 ± 8.3 cm; body mass: 75.1 ± 12.0 kg). A priori power analysis was conducted using G*Power (version 3.1, *α* = 0.05, *β* = 0.80). The analysis revealed that a minimum sample size of 23 participants would be required to detect a moderate correlation of *r* = 0.5. Thus, the sample of 25 participants provided sufficient power for detecting significance of a moderate to very strong correlation [34]. Participants were required to be between the ages of 18 and 35 years and to be informally classified as recreationally active, defined as engaging in recreational activities or moderate-intensity physical activity. Participants were excluded if they experienced any musculoskeletal/neurological disorder (≤1 years before the experiment) that may affect the lower limbs, or if they exceeded the dynamometer threshold on the maximal eccentric isokinetic baseline assessment with a strength level exceeding 3225 N. Prior to enrollment, participants were informed about the procedures, potential risks, and purpose of the study. Participants were required to provide written consent by reviewing and signing an informed consent document that was approved by the Utah State University Institutional Review Board (protocol code #11903 approved 29 April 2021).

### 4.2. Procedures

Participants attended two separate sessions. The first visit was the familiarization session and the second visit was the experimental trial (data collection session). The visits were separated by three to four days to provide ample time for mitigating any soreness experienced post-familiarization. During familiarization, participants were familiarized with all equipment and testing procedures in the following order: warm-up, DJs, CMJs, and orientation to dominant leg MIS and MES assessment. Participants first performed a standardized warm-up that consisted of cycling on a stationary ergometer at 50 watts for five minutes, followed by a dynamic stretching routine (≤10 min) [35]. Then, participants performed three practice jump trials of DJs and CMJs, respectively. They were instructed that for a trial to be considered successful, it must meet the following criteria: (1) execution of the vertical jump with maximal effort while maintaining both feet on the force platform; (2) hands to be kept on the waist throughout the jump; (3) landing from the jump or the drop with both feet impacting the force plate at approximately the same time; and (4) completion of the jump without any additional steps, hops, or loss of balance. Following the jumping trials, participants were guided through a session to acclimate to the Eccentron lower-extremity multi-joint dynamometer (BTE Technologies Inc., Hanover, MD, USA). The session required only 50% of the perceived maximal effort to simulate the MIS and MES measurement process. Participants were provided with sufficient rest (≥5 min) in between each testing phase.

Participants were asked to refrain from engaging in fatiguing exercises for 48 h prior to data collection. Data collection started at approximately the same time of day as familiarization (±2 h). Participants performed the same standardized order of warm-up and testing as used in familiarization. Participants then performed three practice trials of the DJ from a drop height of 0.3 m. Participants were instructed to stand still on the raised platform with arms positioned akimbo. Prior to self-initiating a drop to the landing surface, participants were provided with the following verbal cue: “Whenever you’re ready, step forward off the platform with your preferred foot. Upon landing, perform an immediate jump upwards as quickly and as high as possible.” Following practice, participants were then required to perform three successful trials of the DJ for data collection. For the experimental trials, participants were instructed to stand in a static pose for a minimum of 10 s after landing on a tri-axial force plate (Model OR6-WP, Advanced Mechanical Technology Inc., Watertown, MA, USA), which is recommended for obtaining a valid estimate of landing impact velocity following the initial DJ drop [22].

Following the DJ protocol, participants were asked to perform three practice trials of the CMJ. Participants were instructed to stand still with their feet shoulder width apart on the force plate with arms positioned akimbo. Prior to self-initiating the jump, participants were provided with the following verbal cue: “Whenever you’re ready, jump upwards as high as possible.” Upon landing, participants were asked to return to a standing position without stepping and hold static for several seconds. For the DJ and CMJ, trials were separated by a rest period of approximately 1 min. The akimbo position was maintained throughout the duration of each DJ and CMJ trial to minimize the influence of arm swing on jumping performance. For CMJ trials, the depth of countermovement was self-determined by participants. For DJ trials, participants were allowed to adopt their natural landing strategy without the researchers specifying a squat depth requirement for the ECC phase.

After completing the DJ and CMJ protocols, participants progressed to maximal strength testing. Prior to performing three maximal voluntary ISO contractions, participants were instructed to sit in a reclined position with approximately 90° flexion at the hip joint on the Eccentron and to also position their heels at the base of each pedal in front of them. A member of the research team then adjusted the Eccentron seat to ensure that all participants performed the ISO test with their dominant leg’s knee at an angle of 45°. A small wooden block was used to secure the pedal of the dominant leg in a fixed position. After setup, participants performed each trial of maximal voluntary ISO contraction by rapidly and maximally pushing their dominant foot against the pedal. Participants maintained the application of force for approximately 3 s until instructed by the researcher to relax. During the force application, strong verbal encouragement was provided to ensure the exertion of maximal effort and the maintenance of consistent contact with the base of the seat throughout the effort. A 1 min rest was provided between the three attempts.

Following the MIS test, participants remained on the Eccentron for MES testing. Participants were seated on the Eccentron with their feet in the center of the pedal and their heels positioned at the base of each pedal. The seat and pedal alignment were adjusted to ensure a knee angle of 30 degrees in the most extended position, as recommended by the manufacturer. The Eccentron was set such that the pedals cycled toward and away from participants at a speed of 23 rpm in an alternating motion [36]. Participants were instructed to resist the pedal moving toward them with maximal effort and to relax as it moved away. Six trials of maximal voluntary ECC contraction repetitions were performed per leg. During the MES testing period, participants received the same verbal encouragement as for the MIS testing.

### 4.3. Data Analysis

For DJ and CMJ trials, GRF data were sampled at 1000 Hz from the tri-axial force platform. Raw GRF signals were passed through a fourth-order, recursive, low-pass Butterworth filter (50 Hz cutoff frequency). For CMJ data, filtered GRF signals were sectioned into unloading, ECC, and CON phases [37]. DJ data were analyzed using single force platform methods described by McMahon et al. [22]. After accounting for landing impact velocity, filtered DJ GRF signals were converted to vertical velocity using trapezoidal integration. Using time-synced vertical velocity data, filtered DJ GRF signals were then sectioned into ECC and CON phases. For the comprehensive biomechanical analysis of both DJ and CMJ, the highest *JH* was used among the three jumping trials of each. However, DJ data for one of the 25 participants were excluded due to a data error.

For CMJ data, AmorT was defined as the time interval between countermovement initiation and the transition from the ECC to CON phase. For DJ data, AmorT was defined as the time interval between landing impact and the transition from the ECC to CON phase. For CMJ and DJ data, AmorFz was defined as the vertical GRF value corresponding with the AmorT point.

The RSI and RSImod were computed for the DJ and CMJ, respectively. The RSI was estimated as a linear ratio of *JH* to GCT, while the RSImod was estimated as a linear ratio of *JH* to TTT. GCT was defined as the time interval between landing impact and rebound jump take-off. The timing of both events was estimated using the 10,000 N·s^−1^ RFD criteria described previously [21]. TTT was defined as the time interval between countermovement initiation and jump take-off. CMJ take-off was defined using the same RFD criteria described for the DJ, whereas countermovement initiation was defined as the first time point in data where the vertical GRF fell below a threshold of 5 standard deviations from the mean body weight, which was captured by the force plate during static standing prior to CMJ initiation. For both the RSI and RSImod, *JH* was estimated by inputting take-off velocity (*v_t−off_*) into an equation of constant acceleration (See Equation (1)). *v_t−off_* was determined to be the vertical velocity expressed at the start of the jumping flight phase.

(1) $$JH=\frac{v_{t-off}^{2}}{19.62}$$

For CMJ and DJ data, EccW and ConW were estimated by taking the product of average GRF and total displacement during the ECC and CON phases, with displacement estimated from double trapezoidal integration of GRF data. For CMJs, average GRF and total displacement for the ECC phase were estimated from the time interval between countermovement initiation and AmorT, while for the CON phase, they were estimated from the time interval between AmorT and jump take-off. For DJs, average GRF and total displacement were estimated for the ECC phase from the time interval between landing impact and AmorT, while for the CON phase, they were estimated from the time interval between AmorT and rebound jump take-off. For CMJ and DJ data, EccPP and ConPP were estimated by taking the product of GRF and velocity time-series and then identifying the maximal positive and negative power values.

For CMJ and DJ data, PeakFz was defined as the maximum GRF value occurring during contact between the feet and ground, while MeanConFz was defined as the mean GRF occurring during the CON phase of jumping. For CMJ data, UnRFD and EccRFD were estimated as the linear rate of change in GRF occurring during each jumping phase. For DJ data, EccRFD was estimated by calculating the linear rate of change in GRF occurring during the ECC phase. For DJ data, PFR was derived by subtracting the first successive local GRF minimum from PeakFz [32].

For MIS and MES, a data acquisition system (Biopac MP150WSW, Biopac Systems Inc., Santa Barbara, CA, USA) was used to capture raw force signals from the Eccentron. The data were acquired at a rate of 2 KHz and subsequently processed offline using a custom-written software program (LabVIEW 2018, National Instruments, Austin, TX, USA). The raw signal (V) was converted to Newtons (N), using a manufacturer-provided regression equation. The data were filtered through a fourth-order, zero phase shift Butterworth filter, implementing a 50 Hz cutoff. The ISO peak force was calculated as the highest 500 ms epoch across the entire force curve. Similarly, for the ECC peak force, the single highest data point was determined across the dynamic force curve. For both ISO and ECC peak force variables, the highest repetition was used for subsequent analyses. Raw ISO and ECC peak forces were normalized to participant body mass (N × kg^−1^), and both the raw force values and body-mass normalized data were passed on for statistical analysis.

### 4.4. Statistical Analysis

All statistical analyses were conducted separately for the CMJ and the DJ. CMJ and DJ trials that corresponded with the highest JH achieved were selected for analysis. CMJ data from 25 participants were passed through to analysis. We excluded DJ data from one participant due to an inability to rapidly achieve a static standing position post-landing, resulting in DJ data from 24 participants being analyzed. Correlations between MIS and MES, and the metrics of CMJ and DJ performance were estimated using Pearson *r* correlation coefficients. Pearson *r* values were interpreted using the following scale: 0.00–0.19 very weak; 0.20–0.39 weak; 0.40–0.59 moderate; 0.60–0.79 strong; 0.80–1.00 very strong [38]. The statistical significance of Pearson *r* values was determined using critical value tables for the Pearson correlation coefficient. The cutoff for statistical significance was set at α = 0.05. All statistical analyses were performed in R Studio (version 1.1.456).

## 5. Conclusions

The CMJ and DJ are widely recognized and employed tests for evaluating jump performance across research and strength and conditioning fields. Our study is one of the first to provide a detailed examination of the relationship between lower-extremity multi-joint MES and MIS, and the SSC performance. We accomplished this by analyzing correlations between MES and MIS with a comprehensive array of vertical GRF metrics derived from both CMJs and DJs—two distinct jumping techniques—in young adults within a single investigation. Positive correlations were observed between MES and MIS with RSI, RSImod, and JH, underscoring their contribution to overall jump efficiency and explosive power. Additionally, MES and MIS were positively associated with ConW and ConPP, emphasizing their importance in force application during the propulsion phase. Conversely, negative correlations between MES and MIS with EccW and EccPP suggest that stronger individuals exhibit greater neuromuscular efficiency, reducing energy absorption and enhancing energy transfer. Collectively, these findings demonstrate that MES and MIS are critical for optimizing jump performance by improving energy utilization, force application, and control across phases of the SSC. This insight is particularly valuable for practitioners in strength and conditioning, as it provides a more nuanced understanding of how MES and MIS contribute to overall vertical jumping ability. Such knowledge can be directly applied to the development of targeted training programs designed to improve athletic performance.

## Figures and Tables

**Table 1:** Descriptive statistics for absolute and body mass normalized (BN) maximal eccentric strength (MES), isometric strength (MIS), and countermovement jump metrics.

Measure	Mean	SD
MES (N)	2082.48	529.41
BN MES (N·kg^−1^)	28.23	7.60
MIS (N)	2214.19	637.64
BN MIS (N·kg^−1^)	30.04	9.65
JH (m)	0.34	0.10
TTT (s)	0.76	0.12
RSImod	0.46	0.13
UnRFD (N·s^−1^·kg^−1^)	−35.63	16.71
EccRFD (N·s^−1^·kg^−1^)	68.36	22.20
EccW (J·kg^−1^)	−2.65	0.89
EccPP (W·kg^−1^)	13.96	4.04
AmorT (s)	0.49	0.08
AmorFz (N·kg^−1^)	22.75	3.05
MeanConFz (N·kg^−1^)	19.52	2.20
ConW (J·kg^−1^)	8.33	2.15
ConPP (W·kg^−1^)	49.49	9.05
PeakFz (N·kg^−1^)	23.73	3.03

JH: jump height; TTT: time to take-off; RSImod: reactive strength index modified; UnRFD: unloading rate of force development; EccRFD: eccentric rate of force development; EccW: eccentric work; EccPP: eccentric peak power; AmorT: amortization time; AmorFz: amortization force; MeanConFz: mean concentric force; ConW: concentric work; ConPP: concentric peak power; PeakFz: peak force.

**Table 2:** Descriptive statistics for body mass normalized (BN) maximal eccentric strength (MES), isometric strength (MIS), and depth jump metrics.

Measure	Mean	SD
MES (N)	2058.60	526.86
BN MES (N·kg^−1^)	27.60	7.07
MIS (N)	2165.60	602.23
BN MIS (N·kg^−1^)	29.00	8.29
JH (m)	0.30	0.07
GCT (s)	0.39	0.10
RSI	0.82	0.32
EccRFD (N·s^−1^·kg^−1^)	589.81	304.00
EccW (J·kg^−1^)	−5.40	0.87
EccPP (W·kg^−1^)	−84.73	24.68
AmorT (s)	0.19	0.06
AmorFz (N·kg^−1^)	28.20	8.69
MeanConFz (N·kg^−1^)	21.30	3.62
ConW (J·kg^−1^)	6.39	1.30
ConPP (W·kg^−1^)	47.32	11.18
PeakFz (N·kg^−1^)	38.47	8.42
PFR (W·kg^−1^)	12.67	10.15

JH: jump height; GCT: ground contact time; RSI: reactive strength index; EccRFD: eccentric rate of force development; EccW: eccentric work; EccPP: eccentric peak power; AmorT: amortization time; AmortFz: amortization force; MeanConFz: mean concentric force; ConW: concentric work; ConPP: concentric peak power; PeakFz: peak force; PFR: peak force reduction.

**Table 3:** Pearson *r* correlations between body mass normalized (BN) maximal eccentric strength (MES), isometric strength (MIS), and countermovement jump metrics.

Measure	MES (N)	BN MES (N·kg^−1^)	MIS (N)	BN MIS (N·kg^−1^)
JH (m)	0.63 **	0.65 **	0.75 **	0.75 **
TTT (s)	0.46 *	0.29	0.43 *	0.27
RSImod	0.41 *	0.50 **	0.55 *	0.62 **
UnRFD (N·s^−1^·kg^−1^)	0.15	0.15	0.04	0.05
EccRFD (N·s^−1^·kg^−1^)	−0.07	0.18	0.07	0.28
EccW (J·kg^−1^)	−0.56 **	−0.46 *	−0.57 **	−0.45 *
EccPP (W·kg^−1^)	−0.46 *	−0.45 *	−0.51 **	−0.48 *
AmorT (s)	0.50 **	0.34	0.44 *	0.28
AmorFz (N·kg^−1^)	0.34	0.52 **	0.44 *	0.56 **
MeanConFz (N·kg^−1^)	0.20	0.30	0.19	0.28
ConW (J·kg^−1^)	0.61 **	0.59 **	0.71 **	0.68 **
ConPP (W·kg^−1^)	0.57 **	0.61 **	0.68 **	0.71 **
PeakFz (N·kg^−1^)	0.28	0.45 *	0.35	0.47 *

JH: jump height; TTT: time to take-off; RSImod: reactive strength index modified; UnRFD: unloading rate of force development; EccRFD: eccentric rate of force development; EccW: eccentric work; EccPP: eccentric peak power; AmorT: amortization time; AmorFz: amortization force; MeanConFz: mean concentric force; ConW: concentric work; ConPP: concentric peak power; PeakFz: peak force; * *p* ≤ 0.05; ** *p* ≤ 0.01; Pearson *r* values strength: 0.00–0.19 very weak; 0.20–0.39 weak; 0.40–0.59 moderate; 0.60–0.79 strong; 0.80–1.00 very strong.

**Table 4:** Pearson *r* correlations between body mass normalized (BN) maximal eccentric strength (MES), isometric strength (MIS), and depth jump metrics.

Measure	MES (N)	BN MES (N·kg^−1^)	MIS (N)	BN MIS (N·kg^−1^)
JH (m)	0.65 **	0.59 **	0.67 **	0.62 **
GCT (s)	−0.17	−0.10	−0.10	−0.06
RSI	0.50 *	0.40	0.53 **	0.45 *
EccRFD (N·s^−1^·kg^−1^)	−0.20	−0.22	−0.12	−0.10
EccW (J·kg^−1^)	−0.40	−0.62 **	−0.23	−0.42 *
EccPP (W·kg^−1^)	−0.31	−0.46 *	−0.45 *	−0.60 **
AmorT (s)	−0.17	−0.15	−0.09	−0.11
AmorFz (N·kg^−1^)	0.33	0.26	0.35	0.28
MeanConFz (N·kg^−1^)	0.40	0.28	0.40 *	0.31
ConW (J·kg^−1^)	0.58 **	0.58 **	0.59 **	0.58 **
ConPP (W·kg^−1^)	0.54 **	0.44 **	0.60 **	0.51 **
PeakFz (N·kg^−1^)	0.04	−0.01	0.09	0.07
PFR (W·kg^−1^)	−0.24	−0.29	−0.20	−0.22

JH: jump height; GCT: ground contact time; RSI: reactive strength index; EccRFD: eccentric rate of force development; EccW: eccentric work; EccPP: eccentric peak power; AmorT: amortization time; AmortFz: amortization force; MeanConFz: mean concentric force; ConW: concentric work; ConPP: concentric peak power; PeakFz: peak force; PFR: peak force reduction; * *p* ≤ 0.05; ** *p* ≤ 0.01; Pearson *r* values strength: 0.00–0.19 very weak; 0.20–0.39 weak; 0.40–0.59 moderate; 0.60–0.79 strong; 0.80–1.00 very strong.

## Data Availability

The raw data supporting the conclusion of this article will be made available by the authors upon request.
